# Olfactory response is a potential sign of consciousness: electroencephalogram findings

**DOI:** 10.3389/fnins.2023.1187471

**Published:** 2023-05-18

**Authors:** Wanchun Wu, Chengwei Xu, Qimei Liang, Xiaochun Zheng, Qiuyi Xiao, Haili Zhong, Na Chen, Yue Lan, Xiyan Huang, Qiuyou Xie

**Affiliations:** ^1^Joint Research Centre for Disorders of Consciousness, Department of Rehabilitation Medicine, Zhujiang Hospital, Southern Medical University, Guangzhou, Guangdong, China; ^2^Department of Hyperbaric Oxygen, Zhujiang Hospital, Southern Medical University, Guangzhou, Guangdong, China

**Keywords:** disorders of consciousness, olfactory response, electroencephalogram, diagnosis, prognosis

## Abstract

**Objective:**

This study aimed to explore whether olfactory response can be a sign of consciousness and represent higher cognitive processing in patients with disorders of consciousness (DoC) using clinical and electroencephalogram data.

**Methods:**

Twenty-eight patients with DoC [13 vegetative states (*VS*)/unresponsive wakefulness syndrome (UWS) and 15 minimally conscious states (MCS)] were divided into two groups: the presence of olfactory response (ORES) group and the absence of olfactory response (N-ORES) group according to behavioral signs from different odors, i.e., vanillin, decanoic acid, and blank stimuli. We recorded an olfactory task-related electroencephalogram (EEG) and analyzed the relative power and functional connectivity at the whole-brain level in patients with DoC and healthy controls (HCs). After three months, the outcomes of DoC patients were followed up using the coma recovery scale-revised (CRS-R).

**Results:**

A significant relationship was found between olfactory responses and the level of consciousness (*χ*^2^(1) = 6.892, *p* = 0.020). For olfactory EEG, N-ORES patients showed higher theta functional connectivity than ORES patients after stimulation with vanillin (*p* = 0.029; *p* = 0.027). Patients with N-ORES showed lower alpha and beta relative powers than HCs at the group level (*p* = 0.019; *p* = 0.033). After three months, 62.5% (10/16) of the ORES patients recovered consciousness compared to 16.7% (2/12) in the N-ORES group. The presence of olfactory response was significantly associated with an improvement in consciousness (*χ*^2^(1) = 5.882, *p* = 0.023).

**Conclusion:**

Olfactory responses should be considered signs of consciousness. The differences in olfactory processing between DoC patients with and without olfactory responses may be a way to explore the neural correlates of olfactory consciousness in these patients. The olfactory response may help in the assessment of consciousness and may contribute to therapeutic orientation.

## 1. Introduction

Severe brain injuries may lead to varying stages of disorders of consciousness (DoC), such as coma, vegetative state (*VS*)/unresponsive wakefulness syndrome (UWS), minimally conscious state (MCS), and emergence from MCS (EMCS) (Giacino et al., 2002; Laureys et al., 2010). The clinical evaluation of consciousness is mostly dependent on the behavioral responses of DoC patients to external sensory stimuli. Patients with *VS*/UWS recover their arousal but continue to be insensitive to external stimuli and are unaware of themselves and their surroundings. Patients with MCS display nonreflex activities that indicate consciousness. In clinical practice, auditory and visual-based assessments are the most widely used modalities, which are also subscales in the coma recovery scale-revised (CRS-R) (Giacino et al., 2004; Jain and Ramakrishnan, 2020). However, there is no consensus on whether olfactory stimuli can be used for the behavioral evaluation of consciousness.

The olfactory system is unique because it lacks an obligatory thalamic relay that may provide direct conditions for inducing consciousness (Mori et al., 2013). Merrick et al. (2014) believed that the olfactory system could be used to distinguish between conscious and unconscious processing because, in addition to its anatomical characteristics, it has its own phenomenological, cognitive, and neurodynamic properties. The special phenomenon of olfaction is that it does not produce conscious processing when the concentration of odorants is very low or during sensory habituation to odorants (Walla, 2008). The emergence of consciousness in the olfactory system depends on the synchronization of high-frequency oscillations (beta and gamma) (Mori et al., 2013; Yang et al., 2022), that is, the synchronous integration of widely distributed cortical neuron activities. These high-frequency activities appear to be coupled with respiration, which is linked to slow-wave activities (theta and delta) (Fontanini and Bower, 2006; Kay et al., 2009). High and low oscillations play functional roles in olfactory perception. The primary olfactory cortex, the amygdala, is associated with emotions (Rolls, 2015), whereas the olfactory cortex connects to the hippocampus and is associated with memory (Zhou et al., 2021). The emotions involved in experiencing the external environment may persist in patients with DoC (Steinhoff et al., 2015). Emotional and memorial stimuli may potentially distinguish *VS*/UWS from MCS, or evoke patient consciousness. The uniqueness of the olfactory pathway and its functions make it an ideal system for testing consciousness (Keller and Young, 2014).

Central olfactory processing has been reported to show various degrees of preservation in patients with DoC and has a clear relationship with their consciousness (Nigri et al., 2016). Simultaneously, sniff responses induced by olfactory stimuli are highly predictive in *VS*/UWS patients. Some *VS*/UWS patients with sniff responses eventually transition to MCS (Arzi et al., 2020). When given emotional olfactory stimuli, the mean amplitude of skin conductance increased in DoC patients (Luauté et al., 2018). Based on these previous studies, we believe that olfactory stimuli can induce a conscious behavioral response and predict the recovery of consciousness. However, the effects of olfactory responses in patients with DoC have rarely been studied (Jain and Ramakrishnan, 2020). An objective assessment is needed to clearly define the olfactory response based on observations (Wang et al., 2022).

The purpose of this study was to investigate whether the olfactory response is a sign of consciousness and whether it can represent higher cognitive processing in DoC patients, using clinical and electroencephalogram data. We expect that patients with higher levels of consciousness will have clear responses to olfactory stimuli, and the presence or absence of an olfactory response will help predict the recovery of DoC patients.

## 2. Materials and methods

### 2.1. Study design and participants

Twenty-eight patients with DoC were recruited in this study. Thirteen patients were diagnosed with *VS*/UWS and 15 were diagnosed with MCS based on the CRS-R assessment (Giacino et al., 2004) (see Supplementary material for inclusion and exclusion criteria). We investigated the presence of an olfactory response in these patients and divided them into two groups: ORES group (the presence of olfactory response) and N-ORES group (the absence of olfactory response). Next, we collected the olfactory electroencephalogram (EEG) data from each patient along with data on healthy controls (HCs) (see Supplementary material; Supplementary Table S1). Finally, the patients were followed up for 3 months after the assessments. Written informed consent was obtained from the legally authorized representative of the patients. The ethics committee of Zhujiang Hospital approved all aspects of the study.

### 2.2. Behavioral and outcome data

Each patient was assessed at least three times by two experienced raters using the CRS-R. The best result was retained as the behavioral diagnosis. The olfactory response was assessed using vanillin (pleasant odor), decanoic acid (unpleasant odor), and a blank (see Supplementary material). The rating points of olfactory responses were rated according to the Disorders of Consciousness Scale (DOCS) guidelines (Pape et al., 2005): 0 = No Response, 1 = General Response, and 2 = Localized Response. At the group level, we classified patients into the ORES group (i.e., gained a general response to stimuli with two odorants, gained a general response with one stimulus, or gained a localized response with one stimulus) or the N-ORES group (i.e., no response to stimuli with any odorant). Patients were followed up for 3 months by conducting structured telephone interviews using the CRS-R, according to a previous study (Thibaut et al., 2021). The diagnosis of transition to MCS or EMCS in *VS*/UWS patients, based on CRS-R, was defined as improvement, and the diagnosis of transition to EMCS in MCS patients was also defined as an improvement.

We compared ORES and N-ORES patients with the HC group in terms of age and gender using one-way analysis of variance (ANOVA) and chi-square tests. We also compared the etiology and the duration of injury of ORES and N-ORES patients using Fisher’s exact test and independent-sample *t*-test, and for age and gender using independent-sample *t*-test and Fisher’s exact test. Differences between olfactory responses to the three stimuli were analyzed using McNemar’s test. Statistical differences in the presence of olfactory responses between *VS*/UWS and MCS patients were examined using Fisher’s exact test. Statistical differences in clinical improvement between patients with and without olfactory responses were assessed using the chi-square test.

### 2.3. EEG procedure and statistical analysis

#### 2.3.1. Experimental procedure

The olfactory task was performed while the electrophysiological activity was recorded. We placed two pure odorants (vanillin and decanoic acid) and a blank presentation approximately 2 cm in front of the patients’ nostrils. All the odorants have been used in previous studies (Gottfried et al., 2002; Arzi et al., 2020). Two odorants were presented with felt-tip pens, while one unfilled pen served as a blank (Hummel et al., 1997). We used a blank pen as the baseline to exclude the behavioral responses induced by visual stimuli. During the experiment, the odorant and blank pens were randomly presented to the patients for approximately 5 s. Each pen was presented approximately five times with 30 s intervals in a block design. There were two blocks with 2 min intervals. The protocol used was similar to that used in a previous study (Arzi et al., 2020). E-prime 3.0 (Psychology Software Tools Inc., Pittsburgh, PA, USA) was used to design the experiments. The total number of marks were recorded. All the participants received pleasant odor, unpleasant odor, and blank stimulation. The experiment was performed in a quiet room at an ambient temperature of 24°C and stable humidity.

#### 2.3.2. EEG recording and processing

Brain activity was recorded using a 66 channel system (SynAmps2TM 8500; Neuroscan, USA) at a sampling rate of 2,500 Hz, following the International 10–20 System. The signals were amplified by bandpass filtering at a 1,000 Hz direct current. During the experiment, the electrode impedance was kept below 5 kΩ.

EEG preprocessing was conducted using the EEGLAB toolbox (13_0_0b) in MATLAB (version 2013b; MathWorks Inc., Natick, Massachusetts, USA). The EEG data were filtered between 0.5 and 45 Hz and down-sampled to 500 Hz. The EEG signals were segmented into 10 s epochs using the markers. Independent Component Analysis was used to eliminate the artifacts caused by muscle activity and eye movements. Epochs containing obvious artifacts were manually deleted via visual inspection. A semi-automated process was used to exclude epochs with activity exceeding ±100 μV. Artifact-free signals were used as the average reference. And a fixed number of epochs were used for each participant separately to match trial numbers across groups for further analysis.

#### 2.3.3. EEG data analysis

The following frequency bands were used to analyze the EEG power spectra: delta (0.5–4 Hz), theta (4–8 Hz), alpha (8–13 Hz), and beta (13–30 Hz). Mean connectivity at the whole-brain level was also estimated for the frequency bands and for each group using the weighted Phase Lag Index (wPLI) described in a previous study, see Supplementary material (Vinck et al., 2011). Mean relative power of the entire brain was estimated for each band and group. Absolute power was calculated relative to the total power across the entire frequency spectrum for each frequency band.

Statistical analyses were performed using repeated measures analysis of variance (ANOVA) with the group (HCs, ORES, and N-ORES) as the between-subject factor and type of stimulation (pleasant, unpleasant, and blank) as the within-subject factor. *Post hoc* Bonferroni correction for multiple comparisons was performed when statistically significant differences were observed. SPSS version 22.0 was used to conduct the statistical analysis.

## 3. Results

For patients with DoC, the ORES and N-ORES groups did not significantly differ in terms of age, gender, etiology, or time since injury. Age did not differ significantly among ORES, N-ORES, and HCs (*p* = 0.128), and neither did gender (*p* = 0.437). The demographic data of the patients and comparison of ORES and N-ORES behavioral data are reported in Table 1. Table 2 shows the clinical assessment of ORES patients (see Supplementary Table S2 for clinical data of patients with N-ORES).

**Table 1:** Demographic data summary of the patients and comparison of ORES and N-ORES of EEG.

	DoC patients			Behavioral data		
	Whole sample	MCS	*VS*/UWS	ORES	N-ORES	*p*-value
Participants	28	15	13	16	12	-
Age	48.0 ± 13.4	50.2 ± 12.3	45.5 ± 14.6	48.6 ± 13.2	47.2 ± 14.2	*p* = 0.793
Gender (F/M)	10/18	6/9	4/9	6/10	4/8	*p* = 1.000
Etiology (TBI/nTBI)	10/18	7/8	3/10	3/13	3/9	*p* = 1.000
Time since injury in months	5.4 ± 3.5	4.6 ± 2.3	6.2 ± 4.6	5.13 ± 3.5	5.7 ± 3.7	*p* = 0.697

DoC, disorders of consciousness; ORES, presence of olfactory response; N-ORES, absence of olfactory response; SD, standard deviation; TBI, traumatic brain injury; NTBI, non-traumatic brain injury.

**Table 2:** Demographical, clinical, and outcome data of the 16 patients with olfactory response.

Patient No./gender/age (years)	Etiology	Post- injury (month)	CRS-R diagnosis	Vanillin (pleasant)	decanoic acid (unpleasant)	Blank	Outcome at 3 months (CRS-R)
1/M/53	nTBI	1	MCS	LR	LR	NR	EMCS*
2/M/39	TBI	8	MCS	LR	LR	NR	*VS*/UWS
3/F/48	nTBI	4	MCS	LR	LR	NR	EMCS*
4/M/66	nTBI	15	*VS*/UWS	GR	NR	NR	MCS*
5/M/41	TBI	9	MCS	GR	LR	LR	MCS
6/M/38	nTBI	1	*VS*/UWS	LR	LR	LR	MCS*
7/M/41	nTBI	4	*VS*/UWS	LR	NR	NR	EMCS*
8/F/31	TBI	3	MCS	LR	NR	NR	EMCS*
9/M/25	TBI	4	MCS	GR	LR	NR	MCS
10/F/70	nTBI	8	MCS	GR	LR	NR	EMCS*
11/F/61	TBI	5	MCS	GR	NR	NR	MCS
12/F/67	nTBI	6	MCS	GR	LR	NR	EMCS*
13/M/56	nTBI	4	MCS	LR	LR	NR	EMCS*
14/M/52	TBI	2	MCS	GR	GR	NR	MCS
15/M/50	nTBI	5	MCS	NR	LR	NR	MCS
16/F/40	nTBI	3	*VS*/UWS	GR	GR	NR	EMCS*

CRS-R, coma recovery scale-revised; TBI, traumatic brain injury; NTBI, non-traumatic brain injury; NR, No Response, GR, General Response, and LR, Localized Response; *, improvement.

### 3.1. Behavioral and outcome data

**Figure 1:**
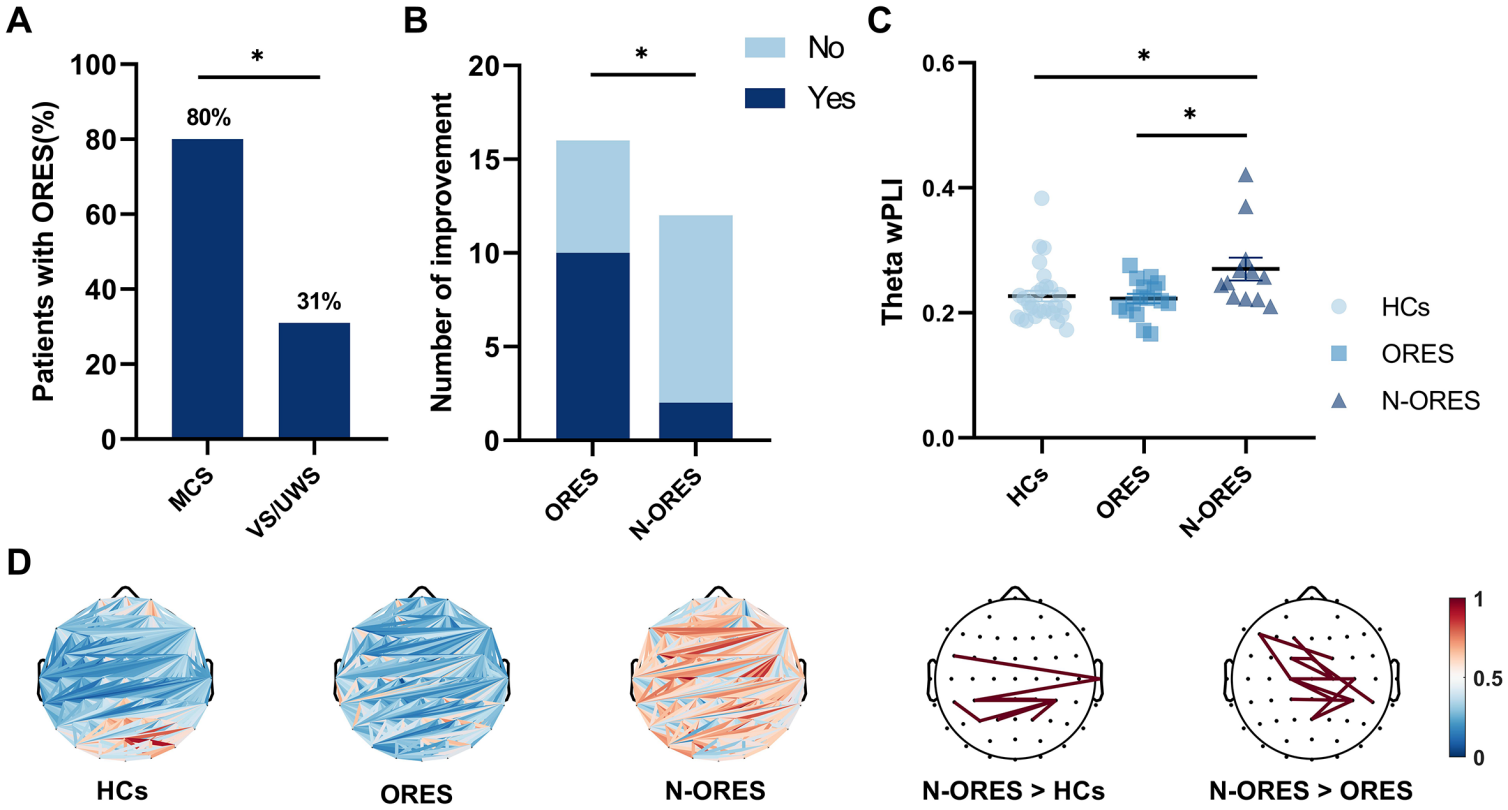
Behavioral results and global connectivity among HCs, ORES, and N-ORES. **(A)** The proportion of olfactory response among MCS and *VS*/UWS patients. A relationship was observed between the presence of olfactory response and level of consciousness (*χ*^2^(1) = 6.892, *p = 0.020). **(B)** The consciousness improvement outcome in the ORES and N-ORES patients (at 3-month follow-up). Patients with olfactory response had higher improvement rates (at 3-month follow-up) than those without response (*χ*^2^(1) = 5.882, *p = 0.023). **(C)** Scatter plot of global wPLI values in theta bands after pleasant stimulus. N-ORES patients showed higher theta connectivity measures compared to ORES patients and HCs (*p = 0.029; *p = 0.027, after Bonferroni correction). **(D)** The top panel shows average connectivity (the first three panels) and significantly altered connectivities in different groups. The red line means significantly increased connectivity (the last two panels).

An olfactory response was present in 16 out of 28 patients (57%), 4 out of 13 patients with *VS*/UWS (31%), and 12 out of 15 patients with MCS (80%). A significant relationship was found between the presence of olfactory response and level of consciousness (*χ*^2^(1) = 6.892, *p* = 0.020, Figure 1A). Among all the patients, 15 showed olfactory responses to pleasant stimuli, 12 showed olfactory responses to unpleasant stimuli, 2 showed olfactory responses to blank stimuli, and 11 patients both showed olfactory response to pleasant stimuli and unpleasant stimuli. When compared to blank stimuli, the incidence of olfactory responses was significantly higher for pleasant and unpleasant stimuli (*χ*^2^(1) = 14.275, *p* = 0.007; *χ*^2^(1) = 9.524, *p* = 0.001). There was no significant difference between the use of pleasant and unpleasant stimuli (*χ*^2^(1) = 0.644, *p* = 1.000). The proportion of traumatic brain injury (TBI) patients (56%) who showed an olfactory response did not differ from nTBI patients (60%) (*χ*^2^(1) = 0.052, *p* = 0.820, Fisher’s exact test: *p* = 1.000).

Outcome data were available for all patients. After three months, 62.5% (10/16) of the ORES patients regained some signs of consciousness compared to 16.7% (2/12) in the N-ORES group. Significant differences in consciousness improvement were found between patients with and without olfactory responses (*χ*^2^(1) = 5.882, *p* = 0.023, Figure 1B).

### 3.2. EEG results

A significant interaction was found between the groups (HCs, ORES, and N-ORES) and stimulations (pleasant, unpleasant, and blank) for functional connectivity in the theta band (*F* = 3.093, *p* = 0.019). No significant main effects were observed in either the group or stimulation. Further interaction analysis indicated that after pleasant stimulation, the N-ORES group showed a higher theta wPLI than the ORES group after Bonferroni correction (*p* = 0.029, Figure 1C). And after pleasant stimulation, the N-ORES group showed a higher theta wPLI than the HCs group after Bonferroni correction (*p* = 0.027, Figure 1C). No significant differences were observed between ORES patients and HCs. Figure 1D showed mean connectivity of the whole brain in three groups after pleasant stimulation.

When observed the difference, the pairwise comparisons of connectivity between every electrode were performed within two groups. The significantly altered connectivity was consistent with increased connectivity. The increased wPLI of the theta band was primarily observed in the central-parietal region in N-ORES patients compared to that in ORES patients and HCs group (Figure 1D, last two panels). There were no significant differences in other frequency bands between the groups and stimulations.

**Figure 2:**
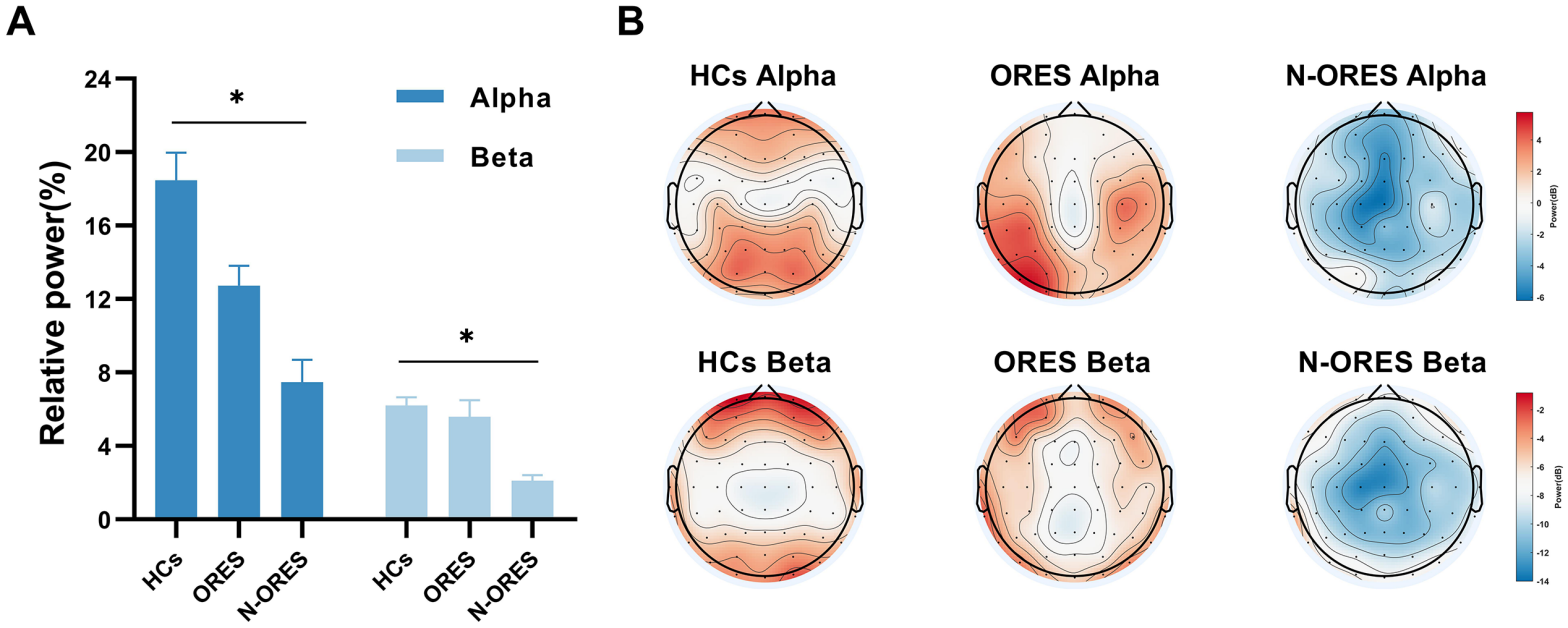
The result of the repeated measures of ANOVA showed that differences in EEG relative power at the group level (HCs, ORES, and N-ORES). **(A)** The relative power in alpha and beta frequency band. A marked decrease in alpha and beta relative power were observed in the N-ORES compared with the power in the HCs group (*p = 0.019; *p = 0.031, after Bonferroni correction). The data were expressed as the means± SEM. **(B)** The power distribution of the whole brain topographic in different groups was displayed.

Power spectral analysis showed a significant main effect for factor ‘group’ in the alpha and beta band (*F* = 4.299, *p* = 0.019 and *F* = 3.634, *p* = 0.033 respectively). Multiple comparisons showed that the N-ORES group had lower alpha and beta relative powers than the HCs group (*p* = 0.019 and *p* = 0.031 respectively, after Bonferroni correction, Figure 2A). Figure 2B showed the power spectra in the top plot between different groups in the alpha and beta bands. No significant interaction or stimulatory effects were observed. The relative power did not differ between the groups and stimulation in other bands.

## 4. Discussion

In the current literature, olfactory stimuli are recommended for assessing the level of consciousness in some scales (Ansell and Keenan, 1989; Rappaport et al., 1992; Gill-Thwaites and Munday, 2004; Pape et al., 2005). However, there is no consensus on whether the olfactory response could be a conscious behavior. Here, we aimed to explore whether the olfactory response is a conscious behavior and reflects a higher level of consciousness (using EEG). We found that the probability of observing an olfactory response increased with the level of consciousness, and that the olfactory response could predict the clinical outcome in patients with DoC. In addition, EEG indicated significant differences between ORES and N-ORES groups. At the whole-brain level, N-ORES patients showed higher theta functional connectivity after pleasant stimuli than ORES patients. N-ORES patients showed lower alpha and beta relative powers than HCs at the group level. Overall, the findings support our hypothesis that olfactory response is a conscious behavior and contributes to research on the importance of consciousness-related olfactory responses.

Clinically, the probability of olfactory responses increased in MCS than *VS*/UWS patients, which is consistent with a previous study that showed that the probability of behavioral response was higher in MCS patients than *VS*/UWS patients (Wang et al., 2022). The presence of an olfactory response in patients with DoC is associated with a higher level of consciousness. This result indicates that olfactory responses could help diagnose the consciousness of patients with DoC. We also showed that the presence or absence of an olfactory response can significantly predict the recovery of consciousness. The ORES patients had a higher improvement rate than the N-ORES patients. This result was inconsistent with that of a previous study in which olfactory behavior could not predict the outcome in DoC patients (Wang et al., 2022). This difference may be due to the different odors used in the study. We chose pure and emotional odors that were more effective and could induce different behaviors (Stevenson et al., 2007; Schriever et al., 2017). We further observed that the proportion of patients with olfactory responses in TBI and nTBI was not significantly different, indicating that the etiology may not affect olfactory function in patients with DoC. However, a previous study showed that patients with TBI and hemorrhage have greater olfactory preservation (Nigri et al., 2016). This inconsistency may be due to different groupings in the research (Marino and Whyte, 2022). In fact, a minority of people have olfactory dysfunction 1 year after TBI (Sigurdardottir et al., 2010). Both pleasant and unpleasant odorants elicited olfactory responses compared to blank. It has been shown that emotions or familiar senses linked to stimuli elicit much stronger responses (Gao et al., 2019; Jain and Ramakrishnan, 2020). Emotional olfactory stimuli may be more effective in awake DoC patients (Martinec Nováková et al., 2021).

At the whole-brain level, N-ORES patients showed a higher theta wPLI than ORES patients after pleasant stimuli. Over the past few years, slow-wave oscillations have been identified as key oscillations associated with olfactory perception and discrimination (Fontanini and Bower, 2006; Kay et al., 2009). Theta oscillations are also locked into the breathing rhythm (Kay, 2014). The association of the olfactory system with many brain regions (Mori et al., 2013) suggests that a robust pathway involving nasal breathing can generate rhythmic electrical activity. There is a distinct reduction in respiratory phase-locked oscillations in theta when the nasal airflow decreases (Zelano et al., 2016; Han et al., 2018). A previous study has shown that when given different olfactory stimuli, most MCS patients had a decrease in nasal airflow volume compared to UWS patients (Arzi et al., 2020). We speculated that the higher theta connectivity in N-ORES patients may be due to their inability to modulate nasal airflow when performing olfactory tasks. Patients with *VS*/UWS do not respond to breathing-based commands (Charland-Verville et al., 2014). Moreover, the level of consciousness may affect olfactory processing. The olfactory bulb receives fewer external inputs under deep anesthesia (Li et al., 2010). Thus, N-ORES patients who have lower consciousness levels might remain in a relatively high-theta connectivity state.

Another interesting finding of our study is the difference caused by pleasantness. Vanilla is a pleasant and familiar odor to the participants in our study. Connectivity differences may arise based on the different valences of the odorants (Callara et al., 2021), whereas motional or hedonic intensities would more strongly influence brain activation (Royet et al., 2003). Emotion involves one’s experiences of external stimuli and is consequently considered “consciousness” (Turner and Knapp, 1995). Emotional stimuli are more likely to attract the attention of patients with DOC (Gao et al., 2019). The association between the limbic system (amygdala and hippocampus) and the primary olfactory cortex is related to emotion and memory in the brain. A previous study demonstrated that the majority of *VS*/UWS patients and all MCS patients showed significant odor-related activation within the amygdala (Nigri et al., 2016). Patients with DoC have various degrees of preservation of the limbic system (Di Perri et al., 2013; Cacciola et al., 2019). This may explain why the pleasant stimuli used in this study were more effective.

Regarding spectral power, the results indicated a lower relative power in the alpha and beta bands at the whole-brain level in N-ORES patients compared to HCs. Lower levels of consciousness have been linked to suppressed alpha activity (Chennu et al., 2014; Rossi Sebastiano et al., 2015). Such configurations in the alpha band are not present in N-ORES patients, demonstrating the importance of alpha power in arousal and awareness. Previous studies have also reported on the role of the alpha band in olfactory tasks. Alpha oscillation is used for concentration, helps classify emotional olfactory stimuli, and is related to odorant administration (Harada et al., 1996; Placidi et al., 2015; Raheel et al., 2019). Lower alpha power in N-ORES patients showed that they could not concentrate well enough to engage in olfactory tasks, even odorless tasks. Beta frequency bands have rarely been considered in patients with DoC (Bai et al., 2020). A previous study reported that lower beta power was present in populations with lower levels of consciousness, representing no thalamocortical activity (Edlow et al., 2021). These results are consistent with our behavioral findings that N-ORES patients have lower consciousness.

Our results suggest that olfactory response should be considered a conscious behavior. We compared the difference between the presence and absence of olfactory responses linked to EEG results and found that theta connectivity may be the neural correlate of olfactory consciousness. The strategy used to identify behavioral correlates of consciousness could relate to the underlying neural mechanisms (Koch et al., 2016). In our study, some *VS*/UWS patients who have olfactory responses transited to MCS or EMCS. If these findings are confirmed in further studies, patients diagnosed with *VS*/UWS who have an olfactory response may be considered as functional MCS (Schnakers et al., 2022). This study also has several limitations. The lack of time control of stimuli is a significant issue (i.e., without using an olfactometer). The olfactometer is a machine that can control the exact timing of olfactory stimuli. However, most olfactometers using large multichannel odorant banks provide limited delivery flexibility and can be expensive to build (Davison and Katz, 2007; Soucy et al., 2009; Tan et al., 2010). Therefore, we particularly analyzed frequency-domain indicators after stimuli to reduce the influence of stimulus time on olfactory perception. It has reported that a single-trail olfactory task without using olfactometer can dynamically reveal changes in hedonic olfactory network (Callara et al., 2021). We hypothesized that such differences would be negligible. Although the number of patients with DoC was limited, we conservatively concluded that some patients with DoC preserved olfactory processing. In addition, future research should follow the recovery of consciousness after olfactory assessments over a longer period. Future research should also include time-frequency indicators or olfactory evoked potentials, which would add to our understanding of olfactory processing.

## 5. Conclusion

Our results confirmed the hypothesis that olfactory response should be considered a sign of consciousness. We observed that olfactory response in patients with DoC had a significant relationship with consciousness level and could predict consciousness recovery. In addition, we observed differences in olfactory processing between patients with and without an olfactory response. Theta connectivity may be a neural correlation with olfactory consciousness in patients with DoC, which could help in the assessment of consciousness and contribute to therapeutic strategies.

## Data availability statement

The original contributions presented in the study are included in the article/Supplementary materials, further inquiries can be directed to the corresponding authors.

## Ethics statement

The studies involving human participants were reviewed and approved by ethics committee of ZhuJiang Hospital. The patients/participants provided their written informed consent to participate in this study.

## Author contributions

WW, QXie, and XH contributed to conception and design of the study. WW, CX, XZ, and QXia organized the experiment. XH, QL, HZ, NC, and YL helped develop the study measures and data collection. WW performed the statistical analysis and wrote the first draft of the manuscript. CX and QL wrote sections of the manuscript. QXie and XH contributed to conceptualization, funding acquisition, resources, supervision, and writing–review and editing. All authors contributed to manuscript revision, read, and approved the submitted version.

## Funding

This study was supported by the National Natural Science Foundation of China (grant Nos. 81974154, 82171174, and 82002374) and Key Scientific and Brain-like research (grant No. 202007030005).

## Conflict of interest

The authors declare that the research was conducted in the absence of any commercial or financial relationships that could be construed as a potential conflict of interest.

## Publisher’s note

All claims expressed in this article are solely those of the authors and do not necessarily represent those of their affiliated organizations, or those of the publisher, the editors and the reviewers. Any product that may be evaluated in this article, or claim that may be made by its manufacturer, is not guaranteed or endorsed by the publisher.

## Abbreviations

DoC: disorders of consciousness
MCS: minimally conscious state
*VS*/UWS: vegetative state/unresponsive wakefulness syndrome
CRS-R: Coma Recovery Scale-Revised
ORES: presence of olfactory response
N-ORES: absence of olfactory response
HCs: healthy controls
DOCS: the Disorders of Consciousness Scale
wPLI: weighted phase lag index
