# Supplementary material for: Olfactory response is a potential sign of consciousness: electroencephalogram findings
Source: Front Neurosci. 2023 May 18;17:1187471. doi: 10.3389/fnins.2023.1187471 (PMC10233028; doi:10.3389/fnins.2023.1187471)
Supplement: Supplementary file 1 [file Table_1.docx]

**Table 1.** Demographical, clinical and outcome data of the N-ORES patients.

| **Patient No./gender/ age(years)** | **Etiology** | **Post-**  **Injury (month)** | **CRS-R Diagnosis** | **Vanillin**  **(pleasant)** | **decanoic acid**  **(unpleasant)** | **Blank** | **Outcome at 3 months（CRS-R）** |
| --- | --- | --- | --- | --- | --- | --- | --- |
| 1/M/44 | nTBI | 12 | VS/UWS | NR | NR | NR | VS/UWS |
| 2/F/33 | nTBI | 1 | VS/UWS | NR | NR | NR | VS/UWS |
| 3/M/32 | nTBI | 5 | VS/UWS | NR | NR | NR | VS/UWS |
| 4/F/67 | nTBI | 4 | VS/UWS | NR | NR | NR | VS/UWS |
| 5/M/58 | TBI | 3 | VS/UWS | NR | NR | LR | VS/UWS |
| 6/M/68 | nTBI | 10 | VS/UWS | NR | NR | LR | VS/UWS |
| 7/M/23 | TBI | 4 | VS/UWS | NR | NR | NR | VS/UWS |
| 8/M/50 | TBI | 5 | MCS | NR | NR | NR | MCS |
| 9/M/44 | TBI | 6 | VS/UWS | NR | NR | NR | VS/UWS |
| 10/F/38 | nTBI | 12 | VS/UWS | NR | NR | NR | VS/UWS |
| 11/M/58 | nTBI | 3 | MCS | NR | NR | NR | EMCS* |
| 12/F/52 | nTBI | 3 | MCS | NR | NR | NR | EMCS* |

CRS-R, coma recovery scale-revised; TBI, traumatic brain injury.TBI, traumatic brain injury; NTBI, non-traumatic brain injury; *, improvement.

**Table 2. Demographical, clinical and outcome data of the healthy controls.**

| **Participant No./gender/ age(years)** | **Pleasantness (V)** | **Arousal**  **(V)** | **Intensity**  **(V)** | **Familiarity**  **(V)** | **Pleasantness**  **(DA)** | **Arousal**  **(DA)** | **Intensity**  **(DA)** | **Familiarity**  **(DA)** |
| --- | --- | --- | --- | --- | --- | --- | --- | --- |
| 1/M/24 | 6.00 | 5.00 | 6.00 | 4.00 | 3.00 | 6.00 | 7.00 | 2.00 |
| 2/M/22 | 7.00 | 7.00 | 7.00 | 6.00 | 4.00 | 7.00 | 7.00 | 4.00 |
| 3/F/26 | 7.00 | 4.00 | 5.00 | 7.00 | 3.00 | 6.00 | 7.00 | 2.00 |
| 4/M/22 | 6.00 | 5.00 | 6.00 | 4.00 | 2.00 | 6.00 | 6.00 | 2.00 |
| 5/M/26 | 4.00 | 5.00 | 5.00 | 2.00 | 8.00 | 7.00 | 4.00 | 5.00 |
| 6/F/22 | 7.00 | 7.00 | 5.00 | 7.00 | 3.00 | 4.00 | 7.00 | 3.00 |
| 7/M/23 | 8.00 | 8.00 | 7.00 | 8.00 | 3.00 | 6.00 | 6.00 | 2.00 |
| 8/F/23 | 7.00 | 5.00 | 6.00 | 5.00 | 2.00 | 5.00 | 7.00 | 1.00 |
| 9/F/23 | 5.00 | 3.00 | 3.00 | 6.00 | 4.00 | 6.00 | 6.00 | 1.00 |
| 10/M/25 | 7.00 | 7.00 | 7.00 | 6.00 | 3.00 | 4.00 | 7.00 | 3.00 |
| 11/M/24 | 8.00 | 7.00 | 7.00 | 7.00 | 4.00 | 5.00 | 6.00 | 3.00 |
| 12/M/25 | 6.00 | 6.00 | 6.00 | 6.00 | 4.00 | 5.00 | 6.00 | 2.00 |
| 13/M/31 | 6.00 | 6.00 | 4.00 | 6.00 | 7.00 | 5.00 | 6.00 | 2.00 |
| 14/M/51 | 6.00 | 6.00 | 6.00 | 4.00 | 3.00 | 7.00 | 7.00 | 4.00 |
| 15/M/48 | 8.00 | 7.00 | 5.00 | 5.00 | 4.00 | 6.00 | 7.00 | 4.00 |
| 16/F/58 | 9.00 | 8.00 | 7.00 | 8.00 | 4.00 | 7.00 | 7.00 | 6.00 |
| 17/M/62 | 6.00 | 5.00 | 6.00 | 6.00 | 4.00 | 5.00 | 5.00 | 6.00 |
| 18/M/52 | 6.00 | 4.00 | 5.00 | 4.00 | 4.00 | 6.00 | 6.00 | 4.00 |
| 19/M/65 | 7.00 | 7.00 | 6.00 | 6.00 | 4.00 | 5.00 | 4.00 | 6.00 |
| 20/F/54 | 5.00 | 5.00 | 5.00 | 6.00 | 4.00 | 5.00 | 6.00 | 4.00 |
| 21/M/58 | 6.00 | 5.00 | 5.00 | 6.00 | 3.00 | 6.00 | 6.00 | 6.00 |
| 22/F/52 | 6.00 | 6.00 | 5.00 | 4.00 | 3.00 | 7.00 | 7.00 | 3.00 |
| 23/F/67 | 6.00 | 5.00 | 6.00 | 7.00 | 4.00 | 5.00 | 5.00 | 4.00 |
| 24/F/50 | 5.00 | 5.00 | 4.00 | 4.00 | 3.00 | 6.00 | 7.00 | 4.00 |
| 25/F/68 | 6.00 | 6.00 | 6.00 | 6.00 | 4.00 | 6.00 | 6.00 | 4.00 |
| 26/F/32 | 8.00 | 7.00 | 6.00 | 7.00 | 4.00 | 6.00 | 6.00 | 2.00 |
| 27/F/33 | 7.00 | 6.00 | 6.00 | 6.00 | 4.00 | 5.00 | 6.00 | 4.00 |
| 28/F/45 | 6.00 | 5.00 | 4.00 | 5.00 | 4.00 | 6.00 | 7.00 | 5.00 |

V, Vanillin; DA, decanoic acid.
