# Supplementary material for: Olfactory response is a potential sign of consciousness: electroencephalogram findings
Source: Front Neurosci. 2023 May 18;17:1187471. doi: 10.3389/fnins.2023.1187471 (PMC10233028; doi:10.3389/fnins.2023.1187471)
Supplement: Supplementary file 2 [file Data_Sheet_1.pdf]

Supplementary Material

1 Inclusion and exclusion criteria

The inclusion criteria were as follows: (1) 18 < age < 70 years old; (2) > 28 days post-injury (prolonged DOC); (3) a diagnosis of VS/UWS or MCS, based on the behavioral assessments using the CRS-R; (4) no tracheostomy, or tracheostomy without obstructive edema of glottis and with decannulation cap and deflate balloons (assessed by laryngoscope or fibrobronchoscope); (5) no history of psychiatric, neurological diseases, or dysosmia; (6) no skull base fracture. The exclusion criteria were as follows: (1) coma or epilepsy; (2) neuromuscular blocking agents or sedative drugs administered within the prior 24 h.

2 Olfactory responses assessment

The olfactory stimuli behaviors were assessed by two raters (well-trained and experienced in DOCS). One rater randomly presented the three odors approximately 2 cm in front of patients′ nostrils and 5 s for each odor. The odor was changed after 30 s, and each odor was given once. One rater recorded behavioral responses. Two independent raters gave the behavioral results according to the recording. They were blind to the patient′s diagnosis. 0 = No Response (NR), no active movement or vocalization following the presentation of stimuli; 1 = Generalized Response (GR), e.g. suckling, jaw movement, chomping/chewing motion, muscle tensing or other movements unrelated to the area stimulated, unrelated vocalizations; 2 = Localized Response (LR), e.g. oral motor movements, such as licking lips or lip compression, tongue pumping or movement.

3 Connectivity analysis

A functional connectivity analysis was performed using the weighted phase-lag index (wPLI). wPLI is defined as the phase difference between two signals, weighted by the magnitude of the imaginary component of the cross-spectrum within the band of interest. The values of wPLI range from 0 to 1, with 1 indicating strong functional connectivity and 0 indicating no connectivity. The mean connectivity of the whole brain was measured for all frequency bands in each group and different types of stimulation. The mean connectivity was used for repeated measures analysis of variance (ANOVA) with the group (HCs, ORES, and N-ORES) and type of stimulation (pleasant, unpleasant, and blank). Post hoc Bonferroni correction for multiple comparisons was performed when statistically significant differences were observed.

When the difference was observed, the pairwise comparisons of connectivity between every electrode were performed using two-sample t-tests, and the network-based statistic was performed after multiple comparisons using the graph theory network analysis toolbox (Wang et al., 2015). An edge p-value of 0.001 was set for the t matrix. Next, statistical significance was estimated with 5,000 permutation tests and a family-wise error corrected level of $P < 0.05$.

4 Materials and results of healthy controls

4.1 Inclusion and exclusion criteria for healthy participants

Healthy volunteers declared themselves healthy with no history of neurological or psychiatric diseases or rhinal disorders. Before inclusion, all healthy participants had to correctly report the onset of both odorants about 5s long per stimulation without prior warning. A false positive or a false negative response would exclude the participant. All healthy participants were able to correctly identify the odor onset. Healthy participants rated the odors for their perceived intensity and pleasantness on a 0-9-point Likert scale at the end of collecting.

4.2 Results

In addition, 28 healthy volunteers (fifteen males; mean age: 39.7 years, SD ± 16.6 years) participated in the EEG portion of the experiment. All healthy participants perceived both odors clearly (vanillin: mean intensity = 5.6, standard deviation (SD) ± 1.0; decanoic acid: mean intensity = 6.2, SD ± 0.9) and rated them as having pleasant and an unpleasant valence (vanillin: mean = 6.5, SD ±1.1; decanoic acid: mean = 3.8, SD ± 1.2). The odors have similar arousal valence (vanillin: mean = 5.8, SD ±1.2; decanoic acid: mean = 5.7, SD ± 0.9) and different familiarity (vanillin: mean = 5.6, SD ± 1.4; decanoic acid: mean = 3.5, SD ± 1.5).

References

Wang, J., Wang, X., Xia, M., Liao, X., Evans, A., and He, Y. (2015). GRETNA: a graph theoretical network analysis toolbox for imaging connectomics. *Front Hum Neurosci* 9, 386. doi: 10.3389/fnhum.2015.00386.
